# The RSNA ImageShare — Enabling Secure, Transparent, and Easy Image Exchange

**DOI:** 10.1007/s10278-021-00537-z

**Published:** 2022-08-22

**Authors:** David S. Mendelson, Chris Carr, Blanca “Didi” Davis

**Affiliations:** 1 Mount Sinai Health System, New York, NY USA; 2 Icahn School of Medicine at Mount Sinai, New York, NY USA; 3 Healthcare Enterprise (IHE) - International, Oak Brook, IL USA; 4 Radiological Society of North America (RSNA), Oak Brook, IL USA; 5 Global Consortium for eHealth Interoperability, Chicago, IL USA; 6 The Sequoia Project, Vienna, VA USA

**Keywords:** Interoperability, RSNA, PHR, Standards, IHE, DICOM

## Abstract

In the early 2000s, the radiology community was awakened to the limitations of electronic media (CDs, DVDs) for exchanging imaging exams. Clinicians frustrated by the time-consuming task of opening discs, while Internet-based exchange of music, photos, and videos were becoming more widespread. The RSNA, which had extensive experience working on interoperability issues in medical imaging, began to look for opportunities to address the issue. In 2007, in the wake of the financial crisis, the National Institute of Biomedical Imaging and Bioengineering (NIBIB) issued an RFP to address Internet-based exchange of medical images. The RFP defined requirements for the network, including that it needed to be patient controlled and standards based. The RSNA was awarded funding for what came to be known as RSNA ImageShare. Over the next 8 years, the RSNA worked in partnership with several vendors and academic institutions to create a network for sharing image-enabled personal health records (PHR). The foundation of interoperability standards used in ImageShare was provided by Integrating the Healthcare Enterprise (IHE), a standards-development organization with which RSNA has had a long association. In 2018 and 2019, the RSNA looked at what had been accomplished and asked if we could take that next step at a national level and promote a solution by which any standards-compliant party could exchange imaging exams through an HIE mechanism.

## Background

“The CD solves the complexities of Image Exchange!” The rationale for the exchange of imaging exams is well established. Briefly, these include [1–4]:

- The need to compare to an historical exam when interpreting a new exam
- Reduction in radiation dose to the patient and general population
- Addressing the high cost of redundant imaging, especially when a recent prior exam is not easily available

In the early 2000s, the radiology community was awakening to the limitations of electronic media (CDs, DVDs) for exchanging imaging exams [1–6]. While an advancement over film, in that discs were able to hold images in digital formats, they employed proprietary formats that often made it difficult and time consuming to view and store the content. Clinicians frustrated by the time-consuming task of opening discs, each with its own novel viewing application, often lined up at radiologists’ offices for help in viewing imaging content. The inconvenience and delay were severe enough to offset the advantages the medium provided and render them impractical as a clinical solution. At the same time, Internet-based exchange of music, photos, and videos was becoming more and more widespread. Many began to question why images and healthcare data in general could not be exchanged over the Internet. Radiologists and consulting physicians began to voice complaints to professional societies, including the RSNA. The RSNA, which already had extensive experience working on interoperability issues in medical imaging, began to look for opportunities to address the issue.

## RSNA ImageShare

In 2007, in the wake of the financial crisis, the National Institute of Biomedical Imaging and Bioengineering (NIBIB) issued an RFP to be financed through the HITECH act [7] to address Internet-based exchange of medical images. The RFP defined requirements for the network, including that it needed to be patient controlled and standards based. The RSNA was awarded funding for what came to be known as RSNA ImageShare. Over the next 8 years, the RSNA worked in partnership with several vendors and academic institutions to create a network for sharing image-enabled personal health records (PHR).

The foundation of interoperability standards used in ImageShare was provided by Integrating the Healthcare Enterprise (IHE), a standards development organization with which RSNA has had a long association. The IHE Cross-Enterprise Document Sharing (XDS) and Cross-Enterprise Document Sharing for Imaging (XDS-I) [8, 9] profiles were the technical specifications employed.

In the first year of the project, the project team defined the architecture and security mechanisms to be put in place and developed an easily deployable device called the Edge Server that implemented these specifications. The Edge Server enabled site personnel to enroll patients and make their imaging data available for secure access.

Two vendors that offer physician- and patient-facing image-enabled PHR systems were contracted for the project. Their systems provided:

- A secure environment to which imaging exams from local radiology systems could be transferred via the Edge Server
- Web browser–based access and viewing of DICOM imaging exams for the patient
- Access to the radiology report
- The ability to download the DICOM images
- The ability for the patient to share browser-based view and download access with care providers via email authentication

Maintaining privacy and data security while enabling web-based patient access was the most challenging aspect of this project. Security and privacy were a shared imperative (and, of course, legal requirement), and the implementation tended toward highly restrictive policies and technical solutions in order to satisfy the requirements of all participating institutions and entities. The implemented solution successfully avoided any data breaches, but it placed obstacles on patients’ participation that likely limited use of the network.

The solution employed avoided the problem of sharing and reconciling patient identifiers across sites that complicates data sharing in the absence of a universal patient identifier in the US health system. Exams were sent directly from a radiology office to the patient’s PHR account, and the patient was provided an alphanumeric security code they could use to access the imaging study and add it to their account.

By 2015 at the close of the first phase of the project, 35,572 patients had enrolled in ImageShare at 20 participating sites across the USA, and 145,672 exams had been distributed.

A survey of patients conducted during the project [10] demonstrated a high level of satisfaction among participants and a preference on the part of these patients for Internet-based exchange over distribution via electronic media. An independent survey conducted by one of the participating sites [11] confirmed a high level of satisfaction with the Internet-based system. An unanticipated finding in this study [11] was the observation that many patients went on to use the system to share their exams with family and friends.

For many patients, the ability to aggregate their exams in a single image-enabled PHR was extremely useful in organizing and expediting their care. Anecdotal comments indicated that patients, often with life-threatening illnesses, benefited greatly from easy access to their healthcare information. Prior to this solution, they often needed to spend weeks gathering dispersed medical records, including their images. As they moved about for consultations and second opinions, care was often delayed as they struggled to collect and share information.

Several vendors have continued to provide image-enabled PHRs following the conclusion of the project up to the present. The vendors have updated their original solutions to address evolving technology.

## Next Steps

The final phase of the NIBIB-funded project, conducted in 2016, addressed the realization that, while the image-enabled PHR was useful for many patients, incorporating medical imaging into an approach more like a traditional health information exchange (HIE) where records of patients who provide consent are made available to care providers without further patient action would expand use of the network.

Such solutions were emerging at a local level, but none on a broader national level. Some regional HIEs included imaging, but again often using proprietary solutions that limit the scope of these networks to a single vendor’s customers. The need to implement standards-based solutions was again recognized as a prerequisite for broad implementation of image exchange.

The RSNA thus explored the best approach for identifying and driving adoption of standards for image exchange, determining that the following steps were needed:

1. Identify the standards.
2. Gain community consensus around the standards.
3. Publish implementation guides for the standards.
4. Establish a means for technology vendors to validate their implementation of the standards

The IHE profiles were again identified as the basis of the proposed solution. Several factors made these attractive. Many elements were in use among HIEs for the exchange of healthcare information other than images. The profiles addressed patient identity, security and authentication, audit trails, and the transport mechanism. The profiles were based upon DICOM 3.0 and HL7 v2.x transactions, both widely deployed. While these profiles are based on aging transaction technologies (ebXML), there is ongoing work to bridge them to standards based on RESTful services, including HL7 FHIR and DICOMweb.

The RSNA Radiology Informatics Committee (RIC) confirmed the choice to make these profiles the foundation for image exchange.

RSNA next sought to partner with an experienced health IT testing entity and established a relationship with the Sequoia Project, an organization building a national infrastructure for healthcare information exchange with a well-developed set of testing resources. RSNA and the Sequoia Project jointly launched the Image Share Validation Program in 2016. Interested health IT vendors were invited to have their products undergo validation testing by Sequoia Project against the defined standards. Successful vendors were awarded a validation seal. Ultimately, nine vendors were validated as part of the 2016 Edition of the validation program. During the initial 2016 edition of the program, some additional areas for improvement were identified. The Image Share Validation program was updated in 2018 to add the robust security requirements and test cases to verify conformance of a system that enabled trust in the data exchanged for all stakeholders.

## Beyond Validation — Exchange in Practice

Throughout the USA, the availability of health information exchange is becoming more widespread, often at a local or state level. The US Office of the National Coordinator for Health Information Technology (ONC) has made interoperability a priority, and the US Center for Medicare & Medicaid Services (CMS) has put rules and regulations behind these efforts [12].

In 2018 and 2019, the RSNA looked at what had been accomplished and asked if we could take that next step at a national level and promote a solution by which any standards-compliant party could exchange imaging exams through an HIE mechanism.

The Sequoia Project [13] had been incubating two initiatives, Carequality [14] and the eHealth Exchange [15], that both support standards-based HIE mechanisms and governance that would enable exchange of healthcare data. These initiatives were growing and gaining success at clinical document exchange, but image exchange was still lacking. In 2018, the Sequoia Project spun off these initiatives into two separate organizations with their own governance structure and leadership.

RSNA expanded their partnership to include Carequality. This partnership that began in early 2019 resulted in an image exchange implementation guide being published and tested to allow the use case to be recognized by the Carequality Steering Committee for production use by implementers in March 2021.

Finally, this allows implementers to deploy a standards-based image exchange use case within their customer base. As a Carequality Connected Agreement (CCA) signee, each implementer agrees to common rules of the road and technical specifications to support various use cases as their technology allows. This removes the need for proprietary point to point interfaces and individual legal agreements that are costly to negotiate. This also paves the way to influence the future requirements that may be imposed by the Trusted Exchange Framework and Connected Agreement (TEFCA) [16]. Carequality is a subcontractor to the Sequoia Project as the Recognized Coordinating Entity (RCE) [17]. Carequality’s role will be to operationalize the deployment of the TEFCA framework and inform future requirements and innovations that will continue to improve interoperability and the exchange of health data.

At the time of this writing (July 2021), there are 5 vendors who have signed the CCA and are currently testing with one another. This is a standard part of the Carequality onboarding process. It serves to identify any gaps or issues present within a vendor’s solution. Through this testing effort, the vendors refine their solutions and ensure that once they go live their interactions with other vendors will be secure and transparent. The 5 current vendors are primarily imaging vendors; however, other vendors in the healthcare domain will be able to consume the imaging exams they expose and ultimately will be enabled to exchange DICOM images that they archive.
